# {5,5′-Bis(diethyl­amino)-2,2′-[(2,2-dimethyl­propane-1,3-di­yl)bis­(nitrilo­methanylyl­idene)]diphenolato}dioxido­molybdenum(VI)

**DOI:** 10.1107/S1600536811035069

**Published:** 2011-09-14

**Authors:** Hadi Kargar, Reza Kia

**Affiliations:** a Chemistry Department, Payame Noor University, Tehran 19395-4697, I. R. of Iran; b X-ray Crystallography Laboratory, Plasma Physics Research Center, Science and Research Branch, Islamic Azad University, Tehran, Iran; c Department of Chemistry, Science and Research Branch, Islamic Azad University, Tehran, Iran

## Abstract

In the title compound, [Mo(C_27_H_38_N_4_O_2_)O_2_], the Mo^VI^ atom is coordinated by two oxide O atoms and by two O and two N atoms of the tetra­dentate Schiff base ligand in a distorted octa­hedral geometry. The Mo—N bond *trans* to a terminal oxide group is significantly longer than the other Mo—N bond, which is attributed to the strong *trans* effect of the oxide O atom. The dihedral angle formed between the substituted benzene rings is 71.79 (14)°. One of the ethyl groups is disordered over two sets of sites, with a refined site-occupancy ratio of 0.588 (18):0.412 (18).

## Related literature

For the chemistry and biochemistry of molybdenum(VI)–Schiff base complexes, see: Enemark *et al.* (2004 ▶); Holm *et al.* (1996 ▶); Mancka & Plass (2007 ▶); Majumdar & Sarkar (2011 ▶). For related structures with MoO_2_ units (metal oxidation state +VI), see: Abbasi *et al.* (2008 ▶); Monadi *et al.* (2009 ▶).
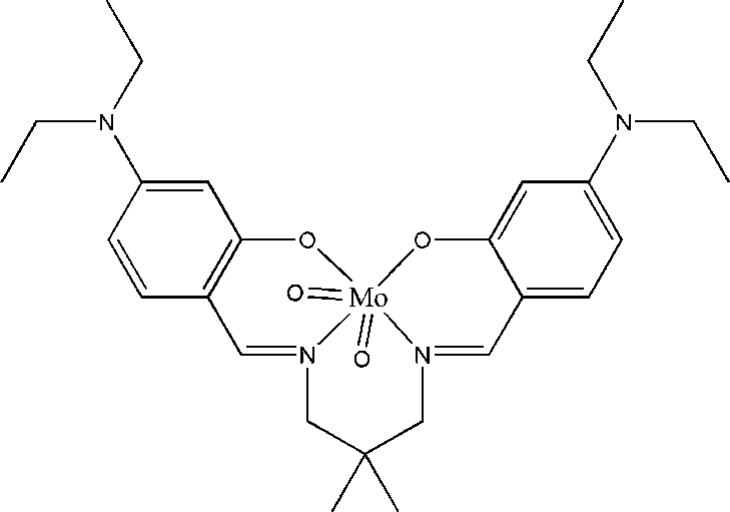


## Experimental

### 

#### Crystal data

[Mo(C_27_H_38_N_4_O_2_)O_2_]
*M* *_r_* = 578.55
Orthorhombic, 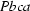

*a* = 9.1561 (9) Å
*b* = 20.6965 (16) Å
*c* = 28.482 (2) Å
*V* = 5397.4 (8) Å^3^
*Z* = 8
Mo *K*α radiation
μ = 0.53 mm^−1^
*T* = 291 K
0.23 × 0.21 × 0.18 mm

#### Data collection

Stoe IPDS 2T Image Plate diffractometer
Absorption correction: multi-scan [*MULABS* (Blessing, 1995 ▶) in *PLATON* (Spek, 2009 ▶)] *T*_min_ = 0.918, *T*_max_ = 1.000
23235 measured reflections
7253 independent reflections
3501 reflections with *I* > 2σ(*I*)
*R*_int_ = 0.083

#### Refinement

*R*[*F* ^2^ > 2σ(*F* ^2^)] = 0.041
*wR*(*F* ^2^) = 0.085
*S* = 0.80
7253 reflections
349 parameters
H-atom parameters constrained
Δρ_max_ = 0.63 e Å^−3^
Δρ_min_ = −0.62 e Å^−3^

### 

Data collection: *X-AREA* (Stoe & Cie, 2009 ▶); cell refinement: *X-AREA*; data reduction: *X-AREA*; program(s) used to solve structure: *SHELXTL* (Sheldrick, 2008 ▶); program(s) used to refine structure: *SHELXTL*; molecular graphics: *SHELXTL*; software used to prepare material for publication: *SHELXTL* and *PLATON* (Spek, 2009 ▶).

## Supplementary Material

Crystal structure: contains datablock(s) global, I. DOI: 10.1107/S1600536811035069/tk2784sup1.cif

Structure factors: contains datablock(s) I. DOI: 10.1107/S1600536811035069/tk2784Isup2.hkl

Additional supplementary materials:  crystallographic information; 3D view; checkCIF report

## Figures and Tables

**Table 1:** Selected bond lengths (Å)

Mo1—O3	1.701 (2)
Mo1—O4	1.710 (2)
Mo1—O2	1.949 (2)
Mo1—O1	2.0875 (18)
Mo1—N1	2.151 (3)
Mo1—N2	2.335 (3)

**Fig. 1.**
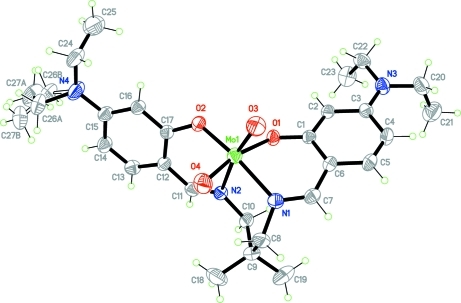
The molecular structure of the title compound, showing 30% probability displacement ellipsoids and the atomic numbering. The atoms with additional labels, A or B, belong to the disordered component of the structure.

## supplementary crystallographic information

### Comment

The element molybdenum is unique among metals due to its varied roles with probably the most prominent role of this element is in the form of bio-catalysts as found in the enzymatic reactions in several molybdoproteins in nature (Majumdar & Sarkar, 2011). The coordination chemistry of molybdenum(VI) has attracted considerable attention due to its biological importance (Enemark *et al.*, 2004; Holm *et al.*, 1996) and their application in various catalytic oxidation reactions (Mancka & Plass, 2007).

In the title compound, Fig. 1, the Mo^VI^ centre is coordinated by two oxide-O atoms and by two O and two N atoms of the tetradentate Schiff base ligand in a distorted octahedral configuration. The dihedral angle between the substituted benzene rings is 71.79 (14) °. The bond lengths and angles are within the normal ranges and comparable to previously reported structures (Abbasi *et al.*, 2008; Monadi *et al.*, 2009). The Mo1—N2 bond length *trans* to the terminal oxido group is significantly longer than the Mo1—N1 bond, a result attributed to the strong *trans* effect of the oxido group (Table 1).

### Experimental

The title complex was prepared by refluxing (3 h) a 1:1 molar ratio of MoO_2_(acac)_2_ and 2,2'-[(2,2-dimethylpropane-1,3-diyl)bis(nitrilomethylidyne)(diethylamino)]dinaphtholate in dry methanol (25 ml). The dark-red crystals were obtained from slow evaporation (several days) of an ethanol solution of the isolated product.

### Refinement

The H atoms were positioned geometrically with C—H = 0.93–0.97 Å and included in a riding model approximation with *U*_iso_(H) = 1.2 or 1.5 *U*_eq_(C). A rotating group model was used for the methyl groups of the diethylamino substituents. One of the ethyl groups was disordered over two positions with a refined site occupancy ratio of 0.588 (18)/0.412 (18).

### Crystal data

**Table:**

[Mo(C_27_H_38_N_4_O_2_)O_2_]	*F*(000) = 2416
*M**_r_* = 578.55	*D*_x_ = 1.424 Mg m^−^^3^
Orthorhombic, *P**b**c**a*	Mo *K*α radiation, λ = 0.71073 Å
Hall symbol: -P 2ac 2ab	Cell parameters from 76.43 reflections
*a* = 9.1561 (9) Å	θ = 1.8–29.5°
*b* = 20.6965 (16) Å	µ = 0.53 mm^−^^1^
*c* = 28.482 (2) Å	*T* = 291 K
*V* = 5397.4 (8) Å^3^	Block, dark-red
*Z* = 8	0.23 × 0.21 × 0.18 mm

### Data collection

**Table:**

Stoe IPDS 2T Image Plate diffractometer	7253 independent reflections
Radiation source: fine-focus sealed tube	3501 reflections with *I* > 2σ(*I*)
graphite	*R*_int_ = 0.083
Detector resolution: 0.15 mm pixels mm^-1^	θ_max_ = 29.2°, θ_min_ = 2.1°
ω scans	*h* = −10→12
Absorption correction: multi-scan [*MULABS* (Blessing, 1995) in *PLATON* (Spek, 2009)]	*k* = −23→28
*T*_min_ = 0.918, *T*_max_ = 1.000	*l* = −38→32
23235 measured reflections	

### Refinement

**Table:**

Refinement on *F*^2^	Primary atom site location: structure-invariant direct methods
Least-squares matrix: full	Secondary atom site location: difference Fourier map
*R*[*F*^2^ > 2σ(*F*^2^)] = 0.041	Hydrogen site location: inferred from neighbouring sites
*wR*(*F*^2^) = 0.085	H-atom parameters constrained
*S* = 0.80	*w* = 1/[σ^2^(*F*_o_^2^) + (0.0298*P*)^2^] where *P* = (*F*_o_^2^ + 2*F*_c_^2^)/3
7253 reflections	(Δ/σ)_max_ = 0.001
349 parameters	Δρ_max_ = 0.63 e Å^−^^3^
0 restraints	Δρ_min_ = −0.62 e Å^−^^3^

### Special details

**Table:**

Geometry. All e.s.d.'s (except the e.s.d. in the dihedral angle between two l.s. planes) are estimated using the full covariance matrix. The cell e.s.d.'s are taken into account individually in the estimation of e.s.d.'s in distances, angles and torsion angles; correlations between e.s.d.'s in cell parameters are only used when they are defined by crystal symmetry. An approximate (isotropic) treatment of cell e.s.d.'s is used for estimating e.s.d.'s involving l.s. planes.
Refinement. Refinement of *F*^2^ against ALL reflections. The weighted *R*-factor *wR* and goodness of fit *S* are based on *F*^2^, conventional *R*-factors *R* are based on *F*, with *F* set to zero for negative *F*^2^. The threshold expression of *F*^2^ > σ(*F*^2^) is used only for calculating *R*-factors(gt) *etc*. and is not relevant to the choice of reflections for refinement. *R*-factors based on *F*^2^ are statistically about twice as large as those based on *F*, and *R*- factors based on ALL data will be even larger.

### Fractional atomic coordinates and isotropic or equivalent isotropic displacement parameters (Å^2^)

**Table:**

	*x*	*y*	*z*	*U*_iso_*/*U*_eq_	Occ. (<1)
Mo1	0.49195 (3)	0.442570 (12)	0.139197 (9)	0.03834 (7)	
O1	0.4476 (2)	0.54138 (9)	0.13559 (7)	0.0380 (5)	
O2	0.3715 (2)	0.43150 (10)	0.08334 (8)	0.0435 (5)	
O3	0.3721 (2)	0.43668 (12)	0.18463 (8)	0.0553 (6)	
O4	0.5773 (2)	0.36911 (10)	0.13823 (10)	0.0583 (6)	
N1	0.6703 (3)	0.47939 (12)	0.18040 (10)	0.0391 (6)	
N2	0.6620 (3)	0.47492 (12)	0.08281 (9)	0.0369 (6)	
N3	0.2751 (3)	0.73949 (13)	0.19983 (10)	0.0462 (7)	
N4	0.2220 (3)	0.30286 (16)	−0.04657 (12)	0.0670 (10)	
C1	0.4504 (3)	0.58292 (14)	0.17016 (11)	0.0332 (7)	
C2	0.3614 (3)	0.63792 (14)	0.16787 (11)	0.0344 (7)	
H2A	0.2977	0.6424	0.1427	0.041*	
C3	0.3648 (3)	0.68669 (15)	0.20226 (11)	0.0371 (7)	
C4	0.4634 (3)	0.67881 (15)	0.24025 (11)	0.0440 (8)	
H4A	0.4677	0.7100	0.2637	0.053*	
C5	0.5515 (3)	0.62622 (16)	0.24259 (11)	0.0437 (8)	
H5A	0.6163	0.6230	0.2676	0.052*	
C6	0.5496 (3)	0.57600 (14)	0.20878 (11)	0.0360 (7)	
C7	0.6614 (3)	0.52931 (16)	0.20827 (11)	0.0413 (8)	
H7A	0.7364	0.5346	0.2300	0.050*	
C8	0.8160 (3)	0.45126 (17)	0.17236 (13)	0.0522 (9)	
H8A	0.8060	0.4052	0.1669	0.063*	
H8B	0.8750	0.4571	0.2003	0.063*	
C9	0.8949 (3)	0.48238 (17)	0.13003 (13)	0.0501 (9)	
C10	0.7854 (3)	0.51603 (15)	0.09690 (12)	0.0434 (8)	
H10A	0.7475	0.5543	0.1124	0.052*	
H10B	0.8368	0.5301	0.0689	0.052*	
C11	0.6523 (3)	0.45916 (14)	0.03940 (12)	0.0391 (8)	
H11A	0.7250	0.4742	0.0194	0.047*	
C12	0.5390 (3)	0.42037 (14)	0.01886 (11)	0.0362 (7)	
C13	0.5568 (3)	0.39713 (16)	−0.02727 (11)	0.0427 (8)	
H13A	0.6415	0.4078	−0.0436	0.051*	
C14	0.4546 (3)	0.35966 (16)	−0.04896 (12)	0.0477 (9)	
H14A	0.4721	0.3445	−0.0792	0.057*	
C15	0.3227 (3)	0.34347 (17)	−0.02622 (12)	0.0432 (8)	
C16	0.2983 (3)	0.37042 (15)	0.01826 (11)	0.0381 (7)	
H16A	0.2091	0.3635	0.0331	0.046*	
C17	0.4042 (3)	0.40730 (14)	0.04073 (11)	0.0350 (7)	
C18	0.9790 (4)	0.4305 (2)	0.10277 (15)	0.0779 (13)	
H18A	1.0274	0.4500	0.0764	0.117*	
H18B	0.9121	0.3982	0.0918	0.117*	
H18C	1.0502	0.4109	0.1230	0.117*	
C19	1.0010 (4)	0.5349 (2)	0.14705 (15)	0.0786 (12)	
H19A	0.9479	0.5672	0.1643	0.118*	
H19B	1.0477	0.5546	0.1205	0.118*	
H19C	1.0735	0.5158	0.1670	0.118*	
C20	0.2832 (4)	0.79287 (17)	0.23365 (14)	0.0596 (10)	
H20A	0.2977	0.7750	0.2648	0.072*	
H20B	0.1906	0.8156	0.2337	0.072*	
C21	0.4011 (5)	0.8398 (2)	0.22395 (16)	0.0779 (13)	
H21A	0.4103	0.8689	0.2500	0.117*	
H21B	0.3780	0.8637	0.1961	0.117*	
H21C	0.4915	0.8171	0.2195	0.117*	
C22	0.1724 (4)	0.74766 (17)	0.16090 (13)	0.0528 (9)	
H22A	0.0958	0.7772	0.1706	0.063*	
H22B	0.1271	0.7063	0.1542	0.063*	
C23	0.2410 (4)	0.77286 (18)	0.11687 (14)	0.0657 (11)	
H23A	0.1680	0.7764	0.0928	0.099*	
H23B	0.3163	0.7437	0.1068	0.099*	
H23C	0.2826	0.8147	0.1228	0.099*	
C24	0.0879 (4)	0.2846 (2)	−0.02313 (14)	0.0633 (11)	
H24A	0.1085	0.2782	0.0100	0.076*	
H24B	0.0553	0.2435	−0.0358	0.076*	
C25	−0.0340 (4)	0.3327 (2)	−0.02772 (16)	0.0821 (14)	
H25A	−0.1194	0.3164	−0.0121	0.123*	
H25B	−0.0554	0.3395	−0.0603	0.123*	
H25C	−0.0053	0.3728	−0.0136	0.123*	
C26A	0.2695 (11)	0.2569 (6)	−0.0854 (3)	0.060 (3)	0.588 (18)
H26A	0.3728	0.2474	−0.0823	0.072*	0.588 (18)
H26B	0.2159	0.2166	−0.0828	0.072*	0.588 (18)
C27A	0.2401 (10)	0.2877 (5)	−0.1326 (4)	0.085 (4)	0.588 (18)
H27A	0.2656	0.2579	−0.1572	0.127*	0.588 (18)
H27B	0.2977	0.3262	−0.1357	0.127*	0.588 (18)
H27C	0.1384	0.2984	−0.1351	0.127*	0.588 (18)
C27B	0.3204 (19)	0.2334 (7)	−0.1047 (8)	0.104 (7)	0.412 (18)
H27D	0.3417	0.2254	−0.1372	0.155*	0.412 (18)
H27E	0.2672	0.1974	−0.0920	0.155*	0.412 (18)
H27F	0.4101	0.2387	−0.0877	0.155*	0.412 (18)
C26B	0.2313 (13)	0.2929 (7)	−0.1004 (6)	0.064 (4)	0.412 (18)
H26C	0.1351	0.2870	−0.1140	0.076*	0.412 (18)
H26D	0.2787	0.3293	−0.1156	0.076*	0.412 (18)

### Atomic displacement parameters (Å^2^)

**Table:**

	*U*^11^	*U*^22^	*U*^33^	*U*^12^	*U*^13^	*U*^23^
Mo1	0.03924 (12)	0.03549 (11)	0.04029 (13)	−0.00411 (14)	0.00207 (15)	−0.00126 (15)
O1	0.0455 (10)	0.0372 (11)	0.0313 (11)	0.0045 (8)	−0.0070 (9)	−0.0051 (10)
O2	0.0376 (11)	0.0498 (14)	0.0430 (13)	−0.0068 (10)	0.0013 (10)	−0.0123 (11)
O3	0.0568 (14)	0.0621 (16)	0.0469 (14)	−0.0094 (13)	0.0114 (11)	0.0036 (14)
O4	0.0646 (15)	0.0350 (12)	0.0753 (17)	−0.0016 (11)	0.0033 (15)	0.0014 (14)
N1	0.0414 (15)	0.0372 (16)	0.0386 (16)	0.0064 (12)	−0.0060 (12)	0.0040 (13)
N2	0.0335 (13)	0.0349 (15)	0.0423 (17)	−0.0025 (11)	−0.0008 (12)	−0.0025 (13)
N3	0.0547 (17)	0.0393 (16)	0.0447 (18)	0.0062 (13)	−0.0016 (14)	−0.0032 (14)
N4	0.056 (2)	0.093 (3)	0.052 (2)	−0.0285 (18)	0.0033 (15)	−0.0278 (19)
C1	0.0314 (14)	0.0345 (15)	0.0338 (17)	−0.0039 (12)	0.0011 (12)	−0.0007 (14)
C2	0.0333 (15)	0.0413 (18)	0.0285 (17)	−0.0002 (13)	−0.0013 (13)	−0.0017 (14)
C3	0.0385 (16)	0.0388 (18)	0.0339 (18)	−0.0004 (14)	0.0032 (13)	0.0012 (15)
C4	0.054 (2)	0.0400 (18)	0.0379 (19)	−0.0007 (15)	−0.0071 (14)	−0.0084 (15)
C5	0.0487 (19)	0.0503 (19)	0.0321 (17)	−0.0063 (15)	−0.0086 (14)	−0.0014 (16)
C6	0.0392 (16)	0.0363 (16)	0.0324 (16)	−0.0031 (12)	−0.0040 (13)	−0.0008 (14)
C7	0.0409 (18)	0.0488 (19)	0.0342 (18)	−0.0032 (15)	−0.0070 (14)	0.0038 (16)
C8	0.0419 (18)	0.047 (2)	0.067 (3)	0.0110 (16)	−0.0086 (17)	−0.004 (2)
C9	0.0333 (16)	0.059 (2)	0.059 (2)	0.0013 (15)	−0.0030 (16)	−0.0093 (19)
C10	0.0398 (17)	0.0400 (18)	0.050 (2)	−0.0094 (14)	0.0000 (15)	−0.0072 (16)
C11	0.0362 (16)	0.0352 (19)	0.046 (2)	−0.0001 (13)	0.0059 (14)	0.0000 (15)
C12	0.0345 (17)	0.0358 (16)	0.0382 (17)	−0.0017 (12)	−0.0039 (13)	−0.0004 (14)
C13	0.0404 (17)	0.053 (2)	0.0343 (18)	−0.0016 (15)	0.0044 (14)	0.0036 (16)
C14	0.051 (2)	0.058 (2)	0.0347 (18)	−0.0049 (16)	−0.0017 (15)	−0.0079 (17)
C15	0.0405 (18)	0.051 (2)	0.038 (2)	−0.0048 (15)	−0.0045 (15)	−0.0028 (17)
C16	0.0358 (16)	0.0417 (18)	0.0368 (19)	−0.0050 (14)	0.0009 (14)	0.0007 (16)
C17	0.0357 (16)	0.0327 (17)	0.0365 (19)	0.0005 (13)	−0.0021 (13)	−0.0018 (14)
C18	0.063 (3)	0.092 (3)	0.079 (3)	0.033 (2)	0.004 (2)	−0.012 (2)
C19	0.060 (2)	0.098 (3)	0.078 (3)	−0.023 (2)	−0.022 (2)	−0.002 (2)
C20	0.064 (3)	0.055 (2)	0.059 (3)	0.0127 (19)	0.0056 (19)	−0.0100 (19)
C21	0.078 (3)	0.064 (3)	0.091 (4)	−0.005 (2)	−0.020 (3)	−0.008 (2)
C22	0.049 (2)	0.043 (2)	0.067 (3)	0.0097 (16)	0.0022 (18)	−0.0001 (19)
C23	0.086 (3)	0.053 (2)	0.059 (3)	−0.005 (2)	−0.003 (2)	0.012 (2)
C24	0.059 (2)	0.071 (3)	0.059 (3)	−0.027 (2)	−0.006 (2)	−0.011 (2)
C25	0.067 (3)	0.103 (4)	0.077 (3)	−0.013 (3)	−0.001 (2)	−0.009 (3)
C26A	0.076 (6)	0.054 (7)	0.049 (6)	−0.018 (5)	−0.006 (4)	−0.008 (5)
C27A	0.094 (6)	0.115 (8)	0.045 (7)	−0.025 (5)	−0.017 (5)	−0.006 (6)
C27B	0.104 (12)	0.076 (10)	0.132 (16)	0.005 (8)	−0.018 (10)	−0.056 (10)
C26B	0.065 (7)	0.072 (9)	0.055 (12)	−0.020 (6)	−0.018 (6)	−0.007 (7)

### Geometric parameters (Å, °)

**Table:**

Mo1—O3	1.701 (2)	C13—H13A	0.9300
Mo1—O4	1.710 (2)	C14—C15	1.410 (4)
Mo1—O2	1.949 (2)	C14—H14A	0.9300
Mo1—O1	2.0875 (18)	C15—C16	1.402 (4)
Mo1—N1	2.151 (3)	C16—C17	1.390 (4)
Mo1—N2	2.335 (3)	C16—H16A	0.9300
O1—C1	1.307 (3)	C18—H18A	0.9600
O2—C17	1.346 (3)	C18—H18B	0.9600
N1—C7	1.305 (4)	C18—H18C	0.9600
N1—C8	1.473 (4)	C19—H19A	0.9600
N2—C11	1.282 (4)	C19—H19B	0.9600
N2—C10	1.470 (4)	C19—H19C	0.9600
N3—C3	1.369 (4)	C20—C21	1.477 (5)
N3—C22	1.464 (4)	C20—H20A	0.9700
N3—C20	1.468 (4)	C20—H20B	0.9700
N4—C15	1.376 (4)	C21—H21A	0.9600
N4—C24	1.448 (4)	C21—H21B	0.9600
N4—C26A	1.521 (11)	C21—H21C	0.9600
N4—C26B	1.550 (17)	C22—C23	1.496 (5)
C1—C2	1.402 (4)	C22—H22A	0.9700
C1—C6	1.434 (4)	C22—H22B	0.9700
C2—C3	1.407 (4)	C23—H23A	0.9600
C2—H2A	0.9300	C23—H23B	0.9600
C3—C4	1.419 (4)	C23—H23C	0.9600
C4—C5	1.356 (4)	C24—C25	1.501 (5)
C4—H4A	0.9300	C24—H24A	0.9700
C5—C6	1.417 (4)	C24—H24B	0.9700
C5—H5A	0.9300	C25—H25A	0.9600
C6—C7	1.408 (4)	C25—H25B	0.9600
C7—H7A	0.9300	C25—H25C	0.9600
C8—C9	1.546 (5)	C26A—C27A	1.514 (16)
C8—H8A	0.9700	C26A—H26A	0.9700
C8—H8B	0.9700	C26A—H26B	0.9700
C9—C18	1.532 (5)	C27A—H27A	0.9600
C9—C19	1.536 (5)	C27A—H27B	0.9600
C9—C10	1.543 (4)	C27A—H27C	0.9600
C10—H10A	0.9700	C27B—C26B	1.48 (2)
C10—H10B	0.9700	C27B—H27D	0.9600
C11—C12	1.436 (4)	C27B—H27E	0.9600
C11—H11A	0.9300	C27B—H27F	0.9600
C12—C13	1.409 (4)	C26B—H26C	0.9700
C12—C17	1.409 (4)	C26B—H26D	0.9700
C13—C14	1.363 (4)		
			
O3—Mo1—O4	104.10 (12)	C13—C14—C15	121.0 (3)
O3—Mo1—O2	104.32 (10)	C13—C14—H14A	119.5
O4—Mo1—O2	98.11 (11)	C15—C14—H14A	119.5
O3—Mo1—O1	88.97 (10)	N4—C15—C16	121.1 (3)
O4—Mo1—O1	163.60 (9)	N4—C15—C14	121.7 (3)
O2—Mo1—O1	87.99 (9)	C16—C15—C14	117.2 (3)
O3—Mo1—N1	95.75 (11)	C17—C16—C15	121.5 (3)
O4—Mo1—N1	88.67 (11)	C17—C16—H16A	119.2
O2—Mo1—N1	156.44 (10)	C15—C16—H16A	119.2
O1—Mo1—N1	80.07 (9)	O2—C17—C16	117.7 (3)
O3—Mo1—N2	166.90 (10)	O2—C17—C12	121.4 (3)
O4—Mo1—N2	86.50 (10)	C16—C17—C12	120.9 (3)
O2—Mo1—N2	81.35 (9)	C9—C18—H18A	109.5
O1—Mo1—N2	79.35 (8)	C9—C18—H18B	109.5
N1—Mo1—N2	76.55 (10)	H18A—C18—H18B	109.5
C1—O1—Mo1	127.10 (19)	C9—C18—H18C	109.5
C17—O2—Mo1	130.82 (18)	H18A—C18—H18C	109.5
C7—N1—C8	117.7 (3)	H18B—C18—H18C	109.5
C7—N1—Mo1	124.4 (2)	C9—C19—H19A	109.5
C8—N1—Mo1	117.6 (2)	C9—C19—H19B	109.5
C11—N2—C10	117.7 (3)	H19A—C19—H19B	109.5
C11—N2—Mo1	123.0 (2)	C9—C19—H19C	109.5
C10—N2—Mo1	119.4 (2)	H19A—C19—H19C	109.5
C3—N3—C22	121.1 (3)	H19B—C19—H19C	109.5
C3—N3—C20	122.5 (3)	N3—C20—C21	114.1 (3)
C22—N3—C20	116.3 (3)	N3—C20—H20A	108.7
C15—N4—C24	122.3 (3)	C21—C20—H20A	108.7
C15—N4—C26A	119.8 (4)	N3—C20—H20B	108.7
C24—N4—C26A	114.4 (4)	C21—C20—H20B	108.7
C15—N4—C26B	117.5 (5)	H20A—C20—H20B	107.6
C24—N4—C26B	117.9 (5)	C20—C21—H21A	109.5
O1—C1—C2	119.2 (3)	C20—C21—H21B	109.5
O1—C1—C6	121.7 (3)	H21A—C21—H21B	109.5
C2—C1—C6	119.0 (3)	C20—C21—H21C	109.5
C1—C2—C3	122.5 (3)	H21A—C21—H21C	109.5
C1—C2—H2A	118.7	H21B—C21—H21C	109.5
C3—C2—H2A	118.7	N3—C22—C23	113.9 (3)
N3—C3—C2	121.6 (3)	N3—C22—H22A	108.8
N3—C3—C4	120.8 (3)	C23—C22—H22A	108.8
C2—C3—C4	117.6 (3)	N3—C22—H22B	108.8
C5—C4—C3	120.5 (3)	C23—C22—H22B	108.8
C5—C4—H4A	119.7	H22A—C22—H22B	107.7
C3—C4—H4A	119.7	C22—C23—H23A	109.5
C4—C5—C6	123.2 (3)	C22—C23—H23B	109.5
C4—C5—H5A	118.4	H23A—C23—H23B	109.5
C6—C5—H5A	118.4	C22—C23—H23C	109.5
C7—C6—C5	120.1 (3)	H23A—C23—H23C	109.5
C7—C6—C1	121.4 (3)	H23B—C23—H23C	109.5
C5—C6—C1	117.1 (3)	N4—C24—C25	114.7 (4)
N1—C7—C6	126.5 (3)	N4—C24—H24A	108.6
N1—C7—H7A	116.7	C25—C24—H24A	108.6
C6—C7—H7A	116.7	N4—C24—H24B	108.6
N1—C8—C9	112.3 (3)	C25—C24—H24B	108.6
N1—C8—H8A	109.1	H24A—C24—H24B	107.6
C9—C8—H8A	109.1	C24—C25—H25A	109.5
N1—C8—H8B	109.1	C24—C25—H25B	109.5
C9—C8—H8B	109.1	H25A—C25—H25B	109.5
H8A—C8—H8B	107.9	C24—C25—H25C	109.5
C18—C9—C19	109.8 (3)	H25A—C25—H25C	109.5
C18—C9—C10	109.4 (3)	H25B—C25—H25C	109.5
C19—C9—C10	106.5 (3)	C27A—C26A—N4	109.4 (11)
C18—C9—C8	109.8 (3)	C27A—C26A—H26A	109.8
C19—C9—C8	110.1 (3)	N4—C26A—H26A	109.8
C10—C9—C8	111.2 (3)	C27A—C26A—H26B	109.8
N2—C10—C9	113.9 (3)	N4—C26A—H26B	109.8
N2—C10—H10A	108.8	H26A—C26A—H26B	108.2
C9—C10—H10A	108.8	C26B—C27B—H27D	109.5
N2—C10—H10B	108.8	C26B—C27B—H27E	109.5
C9—C10—H10B	108.8	H27D—C27B—H27E	109.5
H10A—C10—H10B	107.7	C26B—C27B—H27F	109.5
N2—C11—C12	125.8 (3)	H27D—C27B—H27F	109.5
N2—C11—H11A	117.1	H27E—C27B—H27F	109.5
C12—C11—H11A	117.1	C27B—C26B—N4	102.8 (16)
C13—C12—C17	116.6 (3)	C27B—C26B—H26C	111.2
C13—C12—C11	119.2 (3)	N4—C26B—H26C	111.2
C17—C12—C11	124.1 (3)	C27B—C26B—H26D	111.2
C14—C13—C12	122.5 (3)	N4—C26B—H26D	111.2
C14—C13—H13A	118.7	H26C—C26B—H26D	109.1
C12—C13—H13A	118.7		
			
O3—Mo1—O1—C1	51.0 (2)	C5—C6—C7—N1	177.3 (3)
O4—Mo1—O1—C1	−92.3 (4)	C1—C6—C7—N1	−16.4 (5)
O2—Mo1—O1—C1	155.4 (2)	C7—N1—C8—C9	−89.8 (4)
N1—Mo1—O1—C1	−45.0 (2)	Mo1—N1—C8—C9	83.1 (3)
N2—Mo1—O1—C1	−123.1 (2)	N1—C8—C9—C18	−141.6 (3)
O3—Mo1—O2—C17	−150.9 (3)	N1—C8—C9—C19	97.5 (3)
O4—Mo1—O2—C17	−44.0 (3)	N1—C8—C9—C10	−20.3 (4)
O1—Mo1—O2—C17	120.7 (3)	C11—N2—C10—C9	−114.2 (3)
N1—Mo1—O2—C17	61.5 (4)	Mo1—N2—C10—C9	66.7 (3)
N2—Mo1—O2—C17	41.2 (3)	C18—C9—C10—N2	70.1 (4)
O3—Mo1—N1—C7	−52.2 (3)	C19—C9—C10—N2	−171.2 (3)
O4—Mo1—N1—C7	−156.3 (3)	C8—C9—C10—N2	−51.3 (4)
O2—Mo1—N1—C7	96.3 (3)	C10—N2—C11—C12	−179.0 (3)
O1—Mo1—N1—C7	35.7 (3)	Mo1—N2—C11—C12	0.0 (4)
N2—Mo1—N1—C7	117.0 (3)	N2—C11—C12—C13	−168.3 (3)
O3—Mo1—N1—C8	135.4 (2)	N2—C11—C12—C17	16.4 (5)
O4—Mo1—N1—C8	31.3 (2)	C17—C12—C13—C14	−4.8 (5)
O2—Mo1—N1—C8	−76.1 (3)	C11—C12—C13—C14	179.5 (3)
O1—Mo1—N1—C8	−136.7 (2)	C12—C13—C14—C15	1.5 (5)
N2—Mo1—N1—C8	−55.4 (2)	C24—N4—C15—C16	−1.4 (6)
O3—Mo1—N2—C11	−137.7 (4)	C26A—N4—C15—C16	−159.0 (6)
O4—Mo1—N2—C11	77.8 (3)	C26B—N4—C15—C16	160.8 (7)
O2—Mo1—N2—C11	−20.9 (2)	C24—N4—C15—C14	178.4 (3)
O1—Mo1—N2—C11	−110.5 (2)	C26A—N4—C15—C14	20.8 (7)
N1—Mo1—N2—C11	167.3 (3)	C26B—N4—C15—C14	−19.4 (8)
O3—Mo1—N2—C10	41.3 (5)	C13—C14—C15—N4	−176.4 (3)
O4—Mo1—N2—C10	−103.1 (2)	C13—C14—C15—C16	3.4 (5)
O2—Mo1—N2—C10	158.1 (2)	N4—C15—C16—C17	174.8 (3)
O1—Mo1—N2—C10	68.5 (2)	C14—C15—C16—C17	−5.1 (5)
N1—Mo1—N2—C10	−13.7 (2)	Mo1—O2—C17—C16	142.5 (2)
Mo1—O1—C1—C2	−152.4 (2)	Mo1—O2—C17—C12	−39.5 (4)
Mo1—O1—C1—C6	32.0 (4)	C15—C16—C17—O2	179.8 (3)
O1—C1—C2—C3	−176.1 (3)	C15—C16—C17—C12	1.7 (5)
C6—C1—C2—C3	−0.4 (4)	C13—C12—C17—O2	−174.8 (3)
C22—N3—C3—C2	−0.5 (5)	C11—C12—C17—O2	0.6 (5)
C20—N3—C3—C2	−176.0 (3)	C13—C12—C17—C16	3.2 (4)
C22—N3—C3—C4	−179.5 (3)	C11—C12—C17—C16	178.6 (3)
C20—N3—C3—C4	5.0 (5)	C3—N3—C20—C21	80.7 (4)
C1—C2—C3—N3	−178.8 (3)	C22—N3—C20—C21	−95.0 (4)
C1—C2—C3—C4	0.2 (4)	C3—N3—C22—C23	−79.7 (4)
N3—C3—C4—C5	179.6 (3)	C20—N3—C22—C23	96.1 (4)
C2—C3—C4—C5	0.6 (5)	C15—N4—C24—C25	83.1 (5)
C3—C4—C5—C6	−1.3 (5)	C26A—N4—C24—C25	−118.2 (6)
C4—C5—C6—C7	168.0 (3)	C26B—N4—C24—C25	−79.0 (8)
C4—C5—C6—C1	1.1 (5)	C15—N4—C26A—C27A	−95.0 (7)
O1—C1—C6—C7	8.6 (4)	C24—N4—C26A—C27A	105.7 (6)
C2—C1—C6—C7	−167.0 (3)	C26B—N4—C26A—C27A	1.3 (9)
O1—C1—C6—C5	175.4 (3)	C15—N4—C26B—C27B	94.7 (10)
C2—C1—C6—C5	−0.2 (4)	C24—N4—C26B—C27B	−102.3 (9)
C8—N1—C7—C6	156.9 (3)	C26A—N4—C26B—C27B	−8.8 (10)
Mo1—N1—C7—C6	−15.4 (5)		
